# Rate of Change of Liver Iron Content by MR Imaging Methods: A Comparison Study

**DOI:** 10.3390/tomography8050209

**Published:** 2022-10-08

**Authors:** Shyam Sunder B. Venkatakrishna, Hansel J. Otero, Adarsh Ghosh, Dmitry Khrichenko, Suraj D. Serai

**Affiliations:** 1 Department of Radiology, Children’s Hospital of Philadelphia, 3401 Civic Center Blvd, Philadelphia, PA 19104, USA; 2 Perelman School of Medicine, University of Pennsylvania, 3400 Civic Center Blvd, Philadelphia, PA 19104, USA

**Keywords:** liver iron concentration, LIC, T2 and T2*, R2 and R2*, magnetic resonance imaging

## Abstract

Objective: Magnetic resonance imaging (MRI) can accurately quantify liver iron concentration (LIC), eliminating the need for an invasive liver biopsy. Currently, the most widely used relaxometry methods for iron quantification are R2 and R2*, which are based on T2 and T2* acquisition sequences, respectively. We compared the rate of change of LIC as measured by the R2-based, FDA-approved commercially available third-party software with the rate of change of LIC measured by in-house analysis using R2*-relaxometry-based MR imaging in patients undergoing follow-up MRI scans for liver iron estimation. Methods: We retrospectively included patients who had undergone serial MRIs for liver iron estimation. The MR studies were performed on a 1.5T scanner; standard multi-slice, multi-echo T2- and T2*-based sequences were acquired, and LIC was estimated. The comparison between the rate of change of LIC by R2 and R2* values was performed via correlation coefficients and Bland–Altman difference plots. Results: One hundred and eighty-nine MR abdomen studies for liver iron evaluation from 81 patients (male: 38; female: 43) were included in the study. Fifty-nine patients had two serial scans, eighteen patients had three serial scans, three patients had four serial scans, and one patient had five serial scans. The average time interval between the first and last scans for each patient was 13.3 months. The average rates of change of LIC via R2 and R2* methods were −0.0043 ± 0.0214 and −0.0047 ± 0.012 mg/g per month, respectively. There was no significant difference in the rate of change of LIC observed between the two methods. Linearity between the rate of change of LIC measured by R2 (LIC R2) and R2* (LIC R2*) was strong, showing a correlation coefficient of r = 0.72, *p* < 0.01. A Bland–Altman plot between the rate of change of the two methods showed that the majority of the plotted variables were between two standard deviations. Conclusion: There was no significant difference in the rate of change of LIC detected between the R2 method and the R2* method that uses a gradient echo (GRE) sequence acquired with breath-hold. Since R2* is relatively faster and less prone to motion artifacts, R2*-derived LIC is recommended for iron homeostasis follow-up in patients with liver iron overload.

## 1. Introduction

Repeated blood transfusions, hematological diseases with increased red blood cell turnover, and abnormal homeostasis of iron absorption lead to iron overload states [1,2]. The increased iron content generates reactive oxygen species, which cause tissue damage. Increased liver iron states also result in hepatocyte injury, causing cirrhosis of the liver and hepatocellular carcinoma [3]. Cardiac and endocrine complications can also occur [4,5,6]. Iron overload is commonly seen in children with sickle cell disease, hemochromatosis, thalassemia, congenital anemias, and myelodysplastic syndromes [6]. Although iron overload can be detected using serum ferritin and transferrin levels, these biomarkers can also be abnormal in patients with inflammation, as well as in conditions such as chronic iron deficiency, uremia, malignancy, and nephrotic syndrome [7].

Liver biopsy has historically been the gold standard for monitoring liver iron concentration (LIC), but it is an invasive procedure. Liver biopsy is expensive and is associated with inter- and intra-observer variability, sampling errors, and a low but unignorable complication rate [6,8,9,10]. Magnetic resonance imaging (MRI) is the most widely used non-invasive imaging modality for liver iron quantification and has high sensitivity and specificity [2,11,12,13,14,15,16,17,18]. R2 and R2* calculated from T2 and T2* images are used to estimate LIC [13,15]. A spin-echo-based acquisition sequence is used to calculate R2, whereas a gradient echo (GRE)-based image acquisition sequence is used to calculated R2* [2,6,19]. In this study, we aimed to compare the rate of change of LIC calculated using FDA-approved commercially available third-party software (FerriScan^®^, Resonance Health, Burswood, WA, Australia) with the rate of change of LIC obtained from R2* relaxometry in patients undergoing follow-up serial MRI scans for liver iron estimation.

## 2. Methods

This retrospective Institutional Review Board approved study was conducted in compliance with the Health Insurance Portability and Accountability Act. The need for informed or parental consent was waived. We retrospectively evaluated studies between May 2009 and July 2020 to identify patients who were referred for clinical MRI to evaluate liver iron concentration (LIC). Patients who had both spin-echo imaging and GRE sequences performed for liver iron estimation were included. Patients with incomplete data, non-diagnostic, and/or inadequate technique were excluded.

### 2.1. MR Imaging Protocol

#### 2.1.1. Spin-Echo Imaging (R2 Relaxometry)

The MR images were obtained using a 1.5T scanner (Siemens Healthineers, Malvern, PA, USA). Multi-echo spin-echo axial studies were obtained using a fixed repetition time of 1000 ms, a matrix size of 256, and fields of view between 300 and 400 mm. The slice thickness was set at 5 mm, and increasing echo times (TE) of 6 ms, 9 ms, 12 ms, 15 ms, and 18 ms (spaced at 3 ms intervals) were incorporated in the protocol (Table 1) [2,15,20]. In accordance with FerriScan^®^ protocol guidelines, a one-liter saline bag was placed along the left side of the torso for correction of the external reference to rectify the signal gain changes. The spin-echo images were obtained with only free-breathing, and no compensation techniques were used; with this method, respiratory motion artifact was seen and accepted. Studies with severe motion artifacts that could not be interpreted were excluded. The acquisition scan time took between 12 and 20 min. In the R2 method, there is generation of an R2 map of the liver and application of a calibration curve applied previously for relating R2 values of liver to LIC.

#### 2.1.2. GRE Imaging (R2* Relaxometry)

On the basis of the methodology described by Wood et al., R2* measurements were calculated by obtaining gradient echo multi-echo MR images at increasing TE [13]. All echoes in each repetition time (TR) using an echo train were acquired using this sequence. Eight echo times were used, and the first TE was set as short as technically possible to keep it close to 1 ms. Depending on the selected field of view, 8–10 axial slices were acquired through the liver, and the image acquisition was performed with a 10–12 s single breath-hold. The acquisition parameters of R2* sequence is provided in Table 1.

### 2.2. Post-Processing of Images

For R2-based MR images, the post-processing analysis of images was performed by an external company (FerriScan^®^), and the general principles of their proprietary technique was followed as previously described [15,21,22]. The images were securely sent offsite to Resonance Health for analysis and estimation of LIC values by the R2 method, which was conducted for a fee per study. Mean LIC was calculated using the mean R2 value in the liver with a calibration curve that had been determined through the measurement of liver R2 and needle biopsy LIC [15]. The estimated LIC values were extracted for comparison from the transmitted reports.

For the R2* method, Parametric MRI software (www.parametricmri.com, Philadelphia, PA, USA) developed at our institution was used for post-processing and analysis of images. Individual TE images were used to calculate R2* graphs generated with a mono-exponential fit. Regions of interest (ROI) were drawn on the mid-liver slice of the generated R2* map, and a mean R2* value was calculated [23]. ROIs were drawn in the section of the transverse mid slice that contains the visible liver anatomy, and all vasculature and bile ducts were not included in the ROI. A physician performed the analysis under the supervision of an experienced radiologist and a physicist with a PhD degree and over 15 years of experience in R2* measurement. Using the drawn ROIs, mono-exponential T2* relaxation times were calculated. The mono-exponential T2* relaxation, measured in milliseconds (ms), was calculated using the following equation:

(1) $$S\left(\mathrm{TE}\right)=S0\exp\left(-\frac{\mathrm{TE}}{{T2}^{*}}\right)+C$$

where C is the constant for compensation of contributions from instrument noise and iron-poor species such as bile and blood [24]. The R2* values represented as s^−1^ were obtained using the formula R2* = (1/T2*) × 1000, where T2* was measured in ms. The R2* value was converted to LIC by using the formula LIC = 0.0254 × (R2*) + 0.202 on the basis of the methodology described by Wood et al. (LIC was calculated and measured as mg/g of liver dry weight) [13]. T2*, R2*, and estimated LIC were generated and collected for data analysis and comparisons with changes in R2 and LIC by R2.

The patients with multiple liver MR scans were identified, their LIC values calculated from R2 relaxometry (FerriScan^®^) and R2* relaxometry were reviewed, and the comparison of the rate of change of LIC between these two methods was analyzed.

### 2.3. Statistical Analysis

Continuous variables were reported as mean and standard deviation, while categorical variables were reported as frequencies. The rate of change of LIC was determined as

(2) $$\frac{\Delta \mathrm{LIC}}{\Delta t}=\frac{\mathrm{final}\;\mathrm{LIC}-\mathrm{baseline}\;\mathrm{LIC}}{\mathrm{time}\;\mathrm{interval}\;\mathrm{between}\;\mathrm{the}\;\mathrm{scans}}$$

The relationship between ΔLIC estimated in-house using R2* relaxometry and values calculated by R2 relaxometry were measured using Pearson’s correlation coefficient, intraclass correlation (ICC), and Bland–Altman plots. Significance was set at *p*-value < 0.05, and a correlation coefficient <0.20 was considered very weak; 0.20–0.39, weak; 0.40–0.59, moderate; 0.60–0.79, strong; and ≥0.80, very strong [25]. Statistical analysis was performed with Statistica™ 14.0.0 (TIBCO Software Inc, Palo Alto, CA, USA).

## 3. Results

Eighty-one patients (male: 38; female: 43) with mean age of 13.2 ± 8.3 years (range: 2.9 to 55.7 years) referred for clinical MR liver scans with a total of 189 studies were included in the study. Fifty-nine patients had two serial scans, 18 patients had three serial scans, 3 patients had four serial scans, and 1 patient had five serial scans. The underlying disorders associated with liver iron overload are summarized in Table 2, with beta thalassemia being the most common indication (44 patients). The average time interval between the studies was 13.3 months, with a range of 2.8 to 57.5 months.

The LIC estimated by R2 relaxometry for the 81 subjects ranged from 1.1 to 43.0 mg/g dry tissue, and the LIC calculated by the R2* relaxometry method ranged from 0.4 to 39.1 mg/g dry tissue. These values represent a broad range from normal iron levels to very high iron levels, with 0.17 to 1.8 mg/g dry weight being the reference range for normal iron levels in healthy adults [26].

### 3.1. Estimation of LIC by R2 Relaxometry (LIC R2)

**Figure 1:**
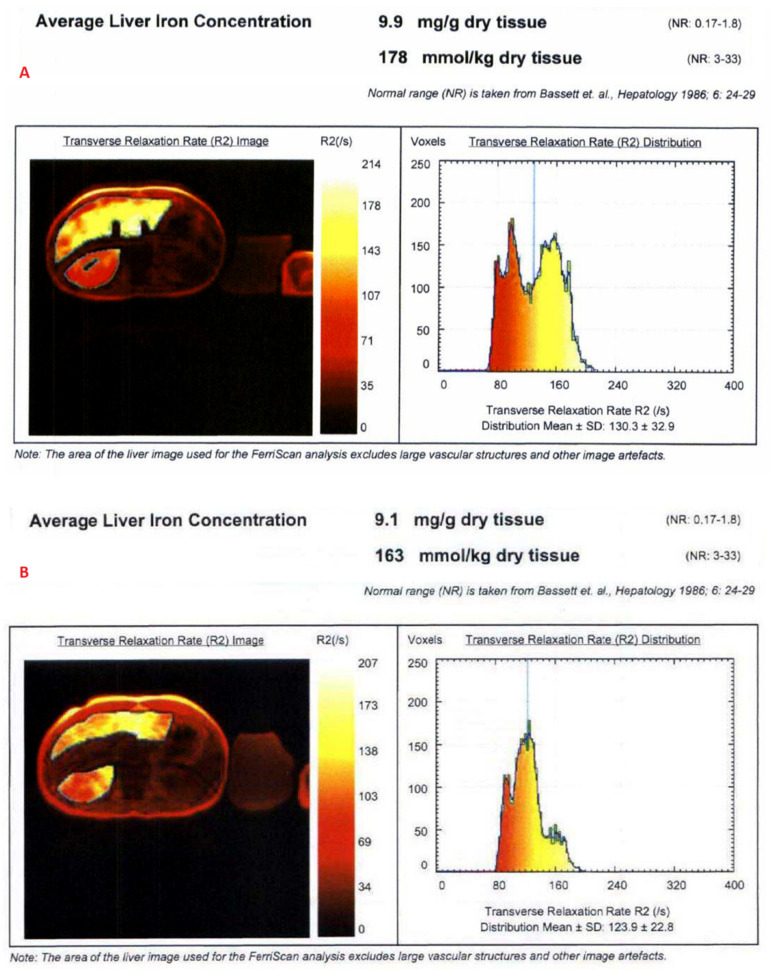

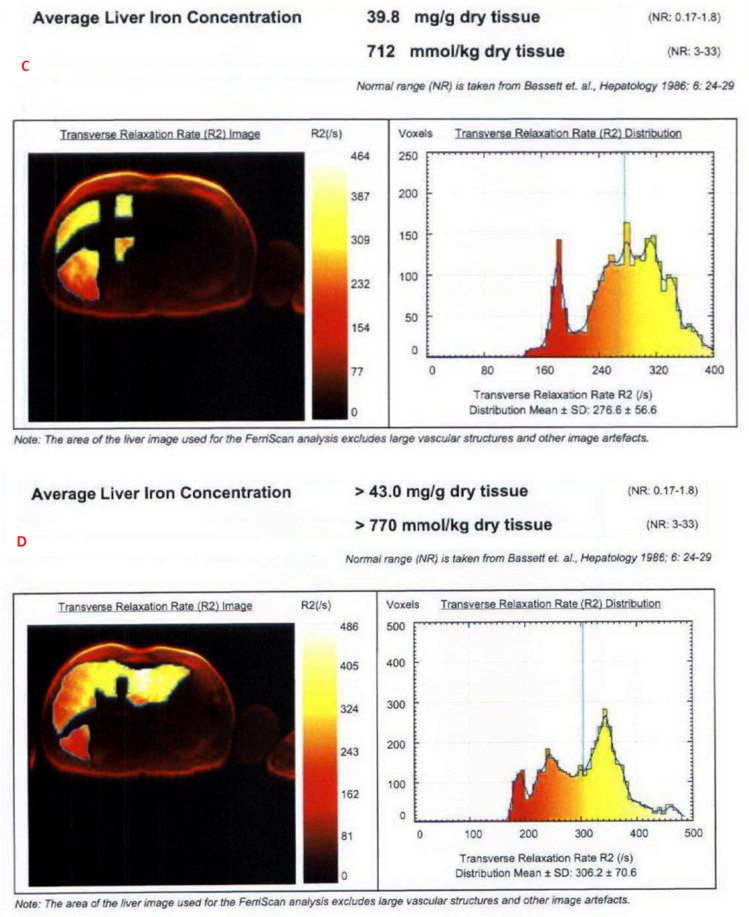
Representative images from anonymized FerriScan^®^ reports of two different patients in the upper (**A**,**B**) and lower panels (**C**,**D**) at two time points (color scale shows the range of R2).

The mean value of LIC R2 at the first scan of the subjects was 14.8 ± 11.9 mg/g (range: 1.1 mg/g to 43 mg/g), and the mean value during last follow-up was 13.2 ± 11.7 mg/g (range: 1.4 mg/g to 43 mg/g). The average rate of change of LIC as determined by R2 (∆LIC R2) was −0.0043 ± 0.0214 mg/g per month. Representative images from anonymized FerriScan^®^ reports of two different patients at two time points are shown in Figure 1. The descriptive statistics for the rate of change of LIC calculated by the two methods are described in Table 3.

### 3.2. Estimation of LIC by R2* Relaxometry (LIC R2*)

**Figure 2:**
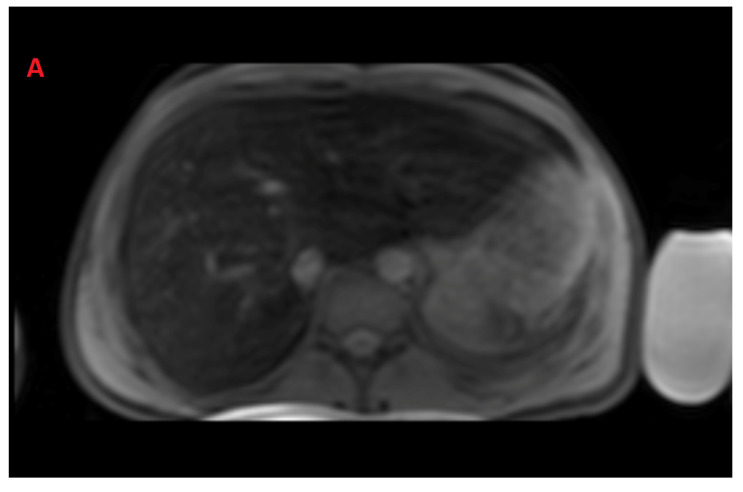

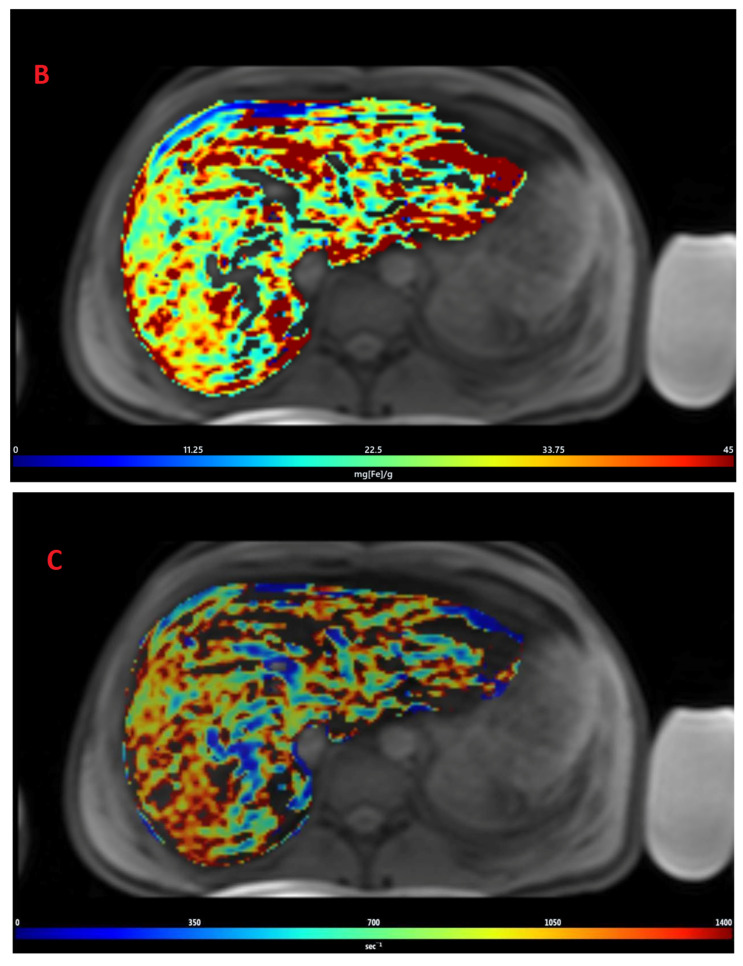

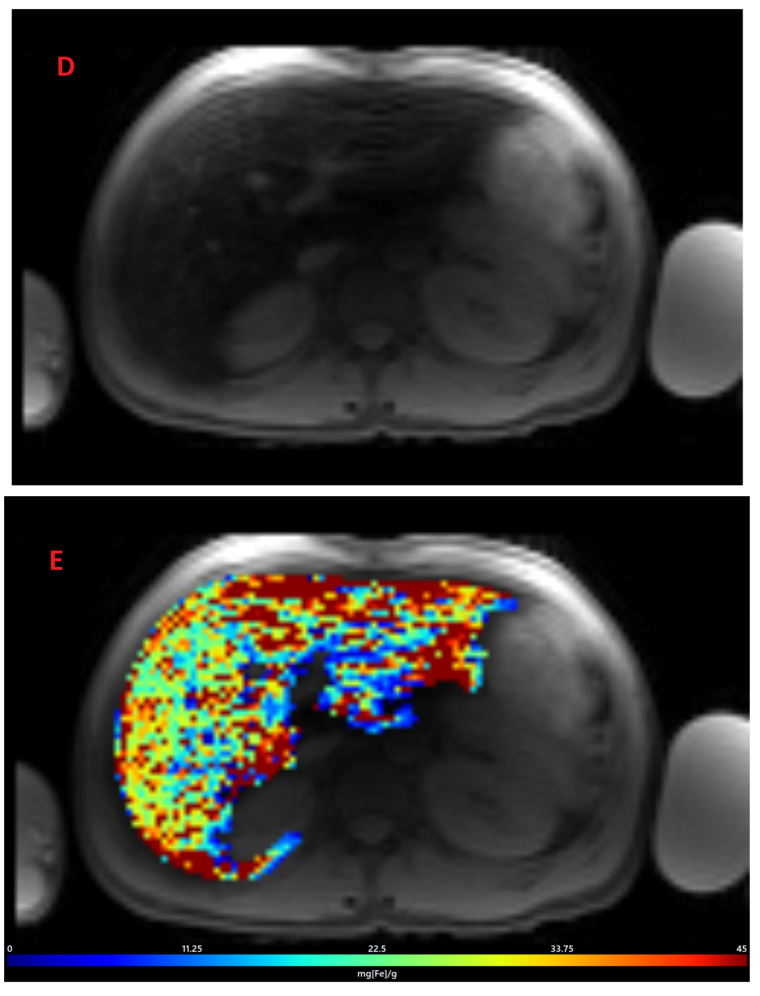

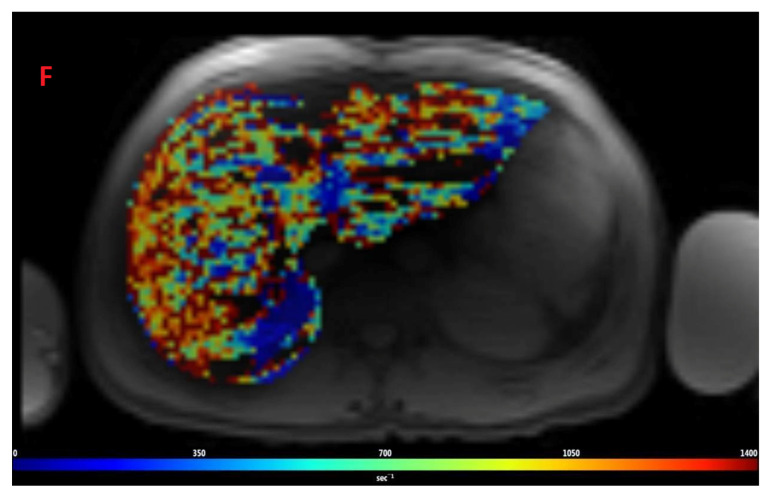
Representative images showing (**A**) the gradient axial echo slices; (**B**) post-processed liver iron concentration (LIC) map (color scale shows the range of LIC from 0 to 45 mg/g); and (**C**) R2* map in a patient with beta thalassemia assessed for iron overload at time point 1, which provided an estimated LIC of 26.6 mg/g. The lower panel (**D**–**F**) demonstrates gradient axial echo slice, post-processed LIC map, and R2* map, respectively, acquired from a scan performed after two years. The estimated LIC was 31.1 mg/g at time point 2.

**Figure 3:**
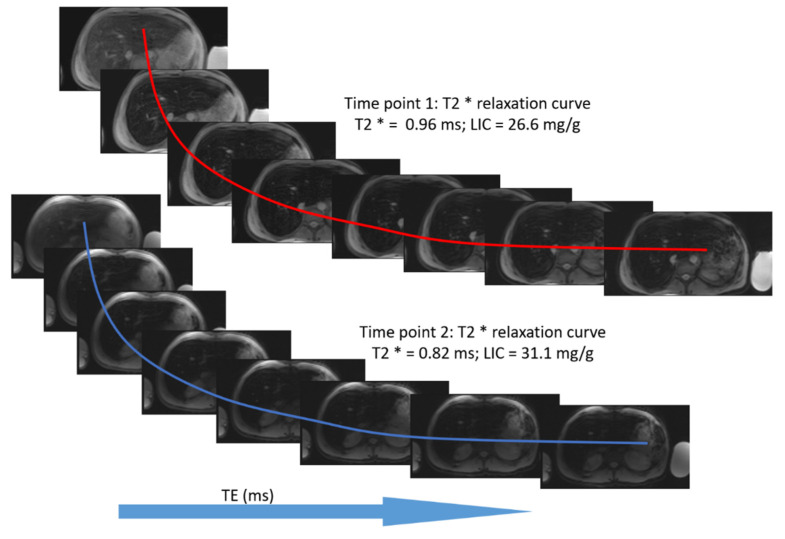
T2* relaxation curve for the same patient at two time points. The second scan was performed approximately two years after the first scan.

The mean value of LIC R2* at the first scan of the subjects was 10.4 ± 7.9 mg/g (range: 1.35 mg/g to 39.1 mg/g), and the mean value of R2* during the last follow-up was 8.4 ± 6.4 mg/g (range: 0.4 mg/g to 31.1 mg/g). The average rate of change of LIC determined by R2* relaxometry (∆LIC R2*) was −0.0047 ± 0.012 mg/g per month. Representative images in a patient with beta thalassemia assessed for iron overload, which provided an estimated LIC of 26.6 mg/g at time point 1 and an estimated LIC of 31.1 mg/g at time point 2 two years later, are shown in Figure 2. T2* relaxation curves for the two time points are shown in Figure 3.

### 3.3. Comparison between LIC R2 and LIC R2*

**Figure 4:**
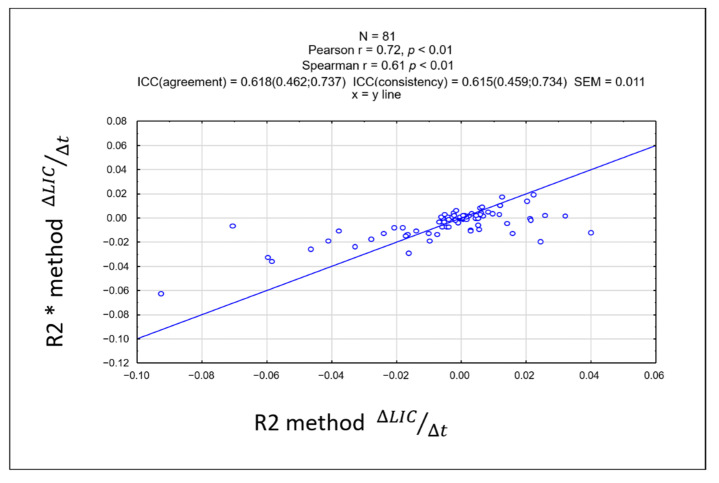
Scatterplot of the rate of change of liver iron concentration (LIC) showing a strong linearity between the R2* and R2-based methods with a correlation coefficient of r = 0.72. This suggests that there were no significant differences in the rate of change of LIC between the two methods.

**Figure 5:**
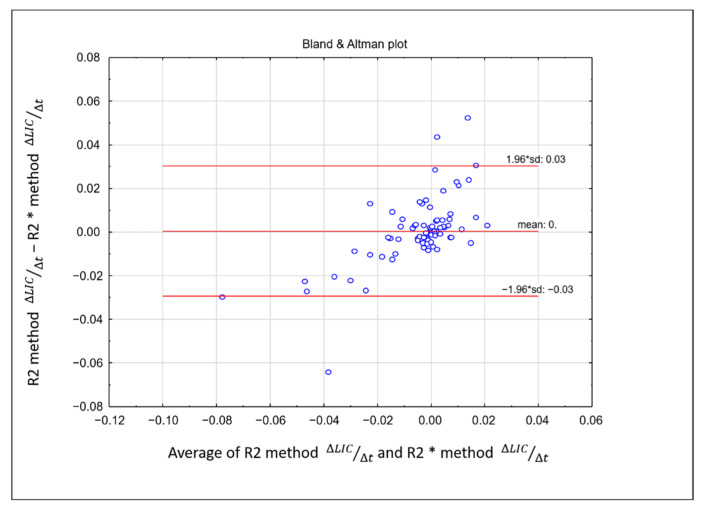
Bland–Altman difference plot demonstrating the difference between the rate of change of liver iron concentration obtained from R2 and R2* methods on the y-axis and the mean of the rate of change from the two methods on the x-axis. The majority of the plotted variables were between the two standard deviations.

There was no significant difference in the rate of change of LIC between the two methods. Linearity between rate of change of ∆LIC R2 and ∆LIC R2* was strong, showing a correlation coefficient of r = 0.72, *p* < 0.01 (Figure 4). The intraclass coefficient (ICC) was 0.618 (95% confidence interval: 0.462–0.737), showing moderate agreement. A Bland-Altman plot between the rate of change of the two methods is shown in Figure 5.

## 4. Discussions

Hepatic fibrosis and cirrhosis, cardiac arrhythmias, diabetes mellitus, and early sudden death are some of the complications of untreated iron overload [27,28]. Monitoring of LIC can assist in determining the timing of chelation therapies, and serial surveillance of LIC can help guide chelation therapy. It is important to note that there was a decrease in the estimated mean values of LIC R2 and LIC R2* from the first scan to the last follow-up scan, as the patients with iron overload may be under treatment with chelation therapy. The estimation of LIC by MRI help the referring doctors in the maintenance or regulation of iron by titration of chelation therapy in such patients. While other studies have compared R2 and R2* relaxometry methods for estimating LIC, our novel evaluation of the rate of change of LIC demonstrates the importance of utilizing this measurement for patients with high liver iron, who often require chelation therapy and may be referred for serial liver iron evaluations. Hence, individual measurements might be less important when compared to the rate of the change in LIC, and this may be the key in the long-term follow-up and management of patients.

Non-invasive modalities for LIC assessment are widely utilized as an alternative to liver biopsy and have been useful in the management of increased iron states in children [29]. T2 and T2* relaxometry estimate liver iron concentration by evaluating signal intensity decay in the liver, with increasing TE using spin-echo and gradient echo images, respectively [6]. This allows for the calculation of liver relaxation rate (R2 or R2*), which is the reciprocal of relaxation time (T2 or T2*, respectively). The relaxation rate has been shown to be directly proportional to LIC. The FerriScan^®^ method, which is based on R2 relaxometry by conventional spin-echo-based imaging, is FDA approved and accepted by many clinical centers for LIC estimation. There are several benefits of the FerriScan^®^ method: the requirements for the MR hardware are less rigorous for analysis of the scans, and there is quality control with annual calibration phantom, which could be useful in centers that have a low volume of these liver scans. Using the FerriScan^®^ method for LIC estimation has limitations: The first is that it requires secure offsite transfer of images to Resonance Health based in Australia for post-processing and analysis, and this leads to a waiting period for the results (2 to 4 business days). There is a fee per study for image analysis, which can be an issue in medical conditions where serial scans are required due to the increased expenses. Usually, a minimum of 15 min is required for a complete FerriScan^®^ image acquisition, so the acquisition time is longer, and the image is therefore prone to motion artifacts. Alternatively, R2*-based LIC measurements can be conducted in a single breath-hold, and the analysis can be performed in-house or by vendor-provided solutions with no delay in reporting values to the referring physician and at no additional cost. In our study, we observed no significant difference between the rate of change of LIC estimated from the R2 relaxometry method and that estimated by the R2* relaxometry method. Therefore, we conclude that LIC can be estimated in-house using the R2* method in conditions that require serial MRI liver scans for LIC assessment and chelation therapy regulation. The R2* relaxometry method is attractive for being non-invasive, and our results confirm that it is as reliable as R2-based LIC assessment. Hence, R2*-relaxometry-based MRI can be used in the accurate, non-invasive assessment of LIC in patients with high iron states and could be particularly valuable for pediatric patients. A comprehensive assessment of the liver can be performed via R2* relaxometry, as 3D imaging of the whole liver in a single breath-hold acquisition time is available [23]. If needed, both liver and cardiac T2* images can also be acquired in a single acquisition [30]. In comparison, FerriScan^®^ takes a relatively longer acquisition time and allows only limited 2D slice imaging [31].

Our study has several limitations that need to be considered. LIC assessment was conducted using all available R2* images at only a single site, and some images were associated with slight motion artifacts, especially in young patients who were unable to follow breath-hold instructions. A free-breathing radial-based acquisition might have the potential to alleviate the need to breath-hold. The ROIs marked in the liver for R2*-relaxometry-based LIC estimation were conducted by a physician. However, with sufficient training and experience, a technologist can easily take over this task. With newer multi-echo Dixon GRE-based acquisition, the R2* map is automatically computed, making the post-processing relatively easier [23]. The procedure of validating non-invasive studies with liver biopsy was not utilized for confirmation of the LIC assessment. However, this did not affect our goal, which was to compare the rate of change of LIC from two independent MRI methods.

## 5. Conclusions

There was no significant difference between the rate of change of liver iron concentration detected using the R2 method and that detected by R2* using a GRE sequence acquired with breath-hold. GRE is advantageous because it is faster and less prone to artifacts. R2 and R2* are not interchangeable for specific time points. However, rate of change and trend are similar, so either method can be used consistently in individual patients.

## Figures and Tables

**Table 1:** Acquisition of R2 and R2* sequence parameters.

	R2 Method	R2* Method
No. of echoes	5	8
Minimum TE (ms)	6	1
Δ TE (ms)	3	1.4
TR (ms)	1000	200
No. of slices	11	4
Slice thickness (mm)	5	10
Matrix size	256 × 256	128 × 128
Flip angle (degree)	90	20
Approx. scan time (minutes/seconds)	10:00	0:13

Key: TE: echo time, ms: time in milliseconds, TR: repetition time, mm: millimeters.

**Table 2:** Table representing the type of disorder associated with the patients referred for clinical MR liver scans.

Diseases	Number (%) Total = 81
Beta thalassemia	44 (54%)
Sickle cell disease	16 (20%)
Hemoglobin H-constant Spring variant	4 (5%)
Diamond–Blackfan anemia	3 (4%)
Idiopathic aplastic anemia	3 (4%)
Pyruvate kinase deficiency	2 (2%)
Aplastic anemia	2 (2%)
Hemoglobin E-beta thalassemia	3 (4%)
Sideroblastic anemia	2 (2%)
Non-autoimmune hemolytic anemia	1 (1%)
Hereditary hemolytic anemia	1 (1%)

**Table 3:** Mean rate of change of liver iron concentration (LIC).

LIC Methods			
	Mean ± SD	Confidence	Confidence
		−95%	95%
Δ LIC R2 (mg/g per month)	−0.0043 ± 0.0214	−0.009037	0.000409
Δ LIC R2* (mg/g per month)	−0.0047 ± 0.012	−0.007384	−0.002108

SD = standard deviation.

## Data Availability

The data presented in this study is available on reasonable request from the corresponding author.
